# PIKfyve inhibitors against SARS-CoV-2 and its variants including Omicron

**DOI:** 10.1038/s41392-022-01025-8

**Published:** 2022-05-24

**Authors:** Jingyi Su, Jing Zheng, Wei Huang, Yali Zhang, Cairui Lv, Baoding Zhang, Lina Jiang, Tong Cheng, Quan Yuan, Ningshao Xia, Jianming Zhang, Li Li, Li Li, Xianming Deng

**Affiliations:** 1 State Key Laboratory of Cellular Stress Biology, Innovation Center for Cell Signaling Network, School of Life Sciences, Xiamen University, 361102 Xiamen, Fujian China; 2 Xiamen Center for Disease Control and Prevention, 361021 Xiamen, Fujian China; 3 State Key laboratory of Molecular Vaccinology and Molecular Diagnostics, National Institute of Diagnostics and Vaccine Development in Infectious Diseases, School of Public Health, Xiamen University, 361102 Xiamen, Fujian China; 4 National Translational Research Center, Ruijin Hospital, Shanghai Jiaotong University School of Medicine, Shanghai, China

**Keywords:** Drug development, Infectious diseases

**Dear Editor**,

COVID-19 pandemic, caused by SARS-CoV-2 infection, is raging around the world and results in millions of deaths since the end of 2019. Although various therapies including vaccines and neutralizing antibodies have been developed to defend against the horrible pandemic, current strategies are inevitably at risk of failure due to high mutagenicity of the viral genome. In fact, the most worrying situation is that the monoclonal antibodies of existing vaccines against the rapidly spreading Omicron variant are ineffective.^1^ Facing the great threat posed by COVID-19, there is an urgent need to develop small molecule antiviral drugs. At present, only a few drugs are authorized to treat COVID-19 in emergency medicine clinics. To identify and evaluate molecular target for COVID-19 becomes a top priority for worldwide health professionals.

It has been reported that PIKfyve might be a potential antiviral target.^2^ PIKfyve is a phosphoinositide 5-kinase that synthesizes PtdIns5P and PtdIns(3,5)biphosphate, which in turn regulates endomembrane homeostasis. Apilimod, an established PIKfyve inhibitor, shows a certain effect in blocking the entry of SARS-CoV-2 into host cells.^2^ Although apilimod has entered a clinical trial against the COVID-19 (NCT04446377), the results have not been published yet and might not be satisfactory because of its unexpected low plasma concentration and poor bioavailability shown in previous failed clinical trials in patients with Crohn’s disease and rheumatoid arthritis.^3^ Coincidentally, our previously internal research on cancer methuosis inducers found a series of 2,4-disubstituted-5H-pyrrolo[3,2-d]pyrimidine derivatives as PIKfyve inhibitors which has distinct scaffold compared with apilimod (Fig. 1a). Among them, XMU-MP-7 (cmpd 38), displayed high affinity for PIKfyve with average *K*d of 6.4 nM (Supplementary Fig. S1) and moderate pharmacokinetic property (Supplementary Table. S1). Molecular docking study revealed the binding mode of XMU-MP-7 with PIKfyve (Supplementary Fig. S2). In this study, we aim to evaluate the antiviral activity of XMU-MP-7 against SARS-CoV-2 and its various variants, especially the highly contagious Delta and the heaviest mutated Omicron, in comparison with apilimod and other FDA-approved small molecule drugs for COVID-19 treatment.

**Fig. 1:**
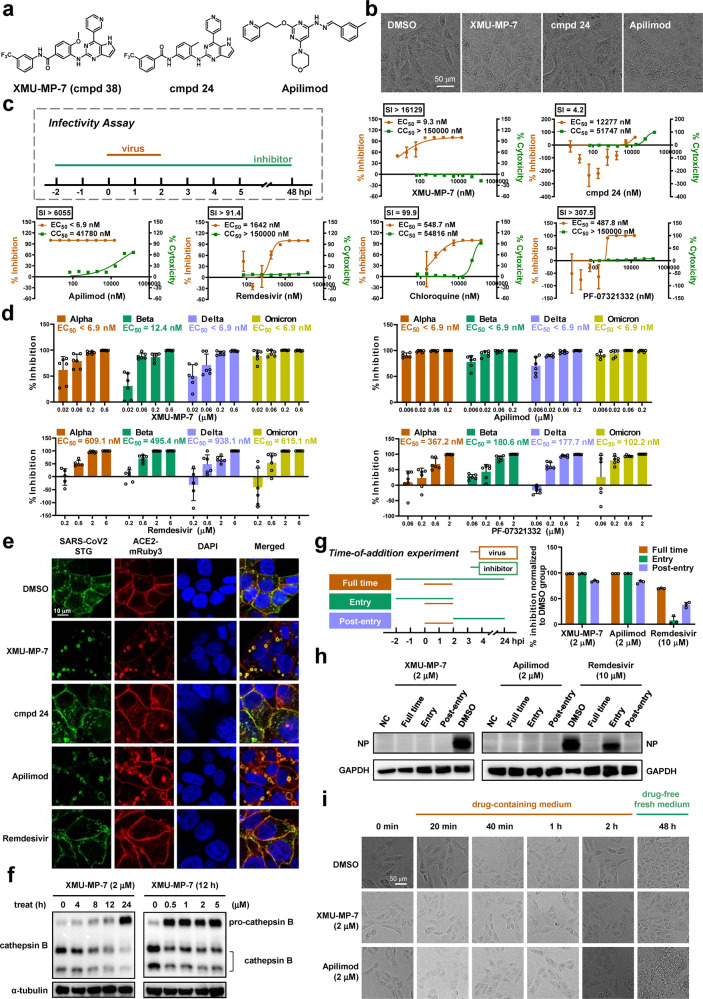
The antiviral efficacy of PIKfyve inhibitors against SARS-CoV-2 and its variants of concern. **a** Structures of PIKfyve inhibitors and a reference cmpd 24. **b** The morphology of Vero E6 cells upon 5 μM drug treatment for 8 h. Scale bar, 50 µm. **c** Schematic of infectivity assay and the antiviral activity of indicated compounds against SARS-CoV-2. Vero E6 cells were infection with SARS-CoV-2 at MOI of 0.05. EC_50_s were determined by qRT-PCR of virus gene (*n* = 3). CC_50_s was determined by MTS assays (*n* = 3). Data are means ± SEM. The Graph is one representative result from three independent experiments. **d** The antiviral ability of PIKfyve inhibitors against four VOCs. Representative data from Fig. S3a. Data were shown as means ± SEM (*n* = 6). **e** Visualization of virus entry using STG probe. Cells were pretreated with 2 μM compounds for 1 h before STG addition. Confocal images of STG (green), ACE2-mRuby3 (red), and nucleus (blue) in 293T-ACE2iRb3 cells were taken at 3 h post STG incubation. Scale bar, 10 µm. **f** XMU-MP-7 inhibits cathepsin B activation. A549 cells were treated with DMSO or XMU-MP-7 for different time or concentrations as indicated. Cathepsin B and α-tubulin were analyzed by Western blot. **g**, **h** Time-of-addition experiment of PIKfyve inhibitors. The specific treatment of drugs and viruses at different stages was shown in the scheme. Viral replication was quantified by qRT-PCR at 24 h post infection (*n* = 3) (**g**). Nucleoprotein and GAPDH were analyzed by Western blot (**h**). NC, negative control, protein sample derived from cells that were not infected with virus. **i** PIKfyve inhibitors induce vacuolization in entry and post-entry stages. Vero E6 cells were treated with 2 μM compounds or DMSO for different time periods. Two hours after treatment, cells were washed twice with PBS and cultured in drug-free medium for another 48 h. Scale bar, 50 µm

Accumulating huge vacuoles in cell cytoplasm is the characteristic of PIKfyve inhibition. As expected, XMU-MP-7 dramatically induced cytoplasmic vacuoles in vero E6 cells as apilimod did. Cpmd 24, an XMU-MP-7 analog without PIKfyve binding affinity (*K*d > 30,000 nM), could not induce visible vacuoles in the same condition (Fig. 1b). We then determined their antiviral activity against SARS-CoV-2 wild-type isolate XM088T, which was highly homologous to the SARS-CoV-2 isolate Wuhan-Hu-1. Vero E6 cells were pretreated with individual drugs for 2 h before infection, and virus released in supernatants was detected via quantitative real-time PCR (qRT-PCR) at 48 h post infection (p.i.) (Fig. 1c). To our surprise, XMU-MP-7 showed the half-maximal effective concentration (EC_50_) of 9.3 nM which was far better than approved antiviral agents, as adenosine analogue Remdesivir showed an EC_50_ of 1642 nM and lysosomal acidification inhibitor Chloroquine had an EC_50_ of about 500 nM. PF-07321332, the key component of the newly approved oral drug PAXLOVID, exhibited an EC_50_ of 487 nM, which is comparable to that of other studies.^4^ Given the extremely low cytotoxicity of XMU-MP-7 (CC_50_ > 150 μM), its selectivity index (SI, CC_50_/EC_50_) was even higher than apilimod. In contrast, cmpd 24, showed a fairly weak antiviral activity (EC_50_ = 12277 nM; SI = 4.21) (Fig. 1c). Taken together, PIKfyve inhibitor XMU-MP-7 exhibits potent antiviral ability against wildtype SARS-CoV-2 in vitro.

We further evaluated the efficacy of the PIKfyve inhibitors against four SARS-CoV-2 variants of concern (VOCs), including Alpha, Beta, Delta and Omicron. Results showed similar drug sensitivity among SARS-CoV-2 wild-type and variants. XMU-MP-7 achieved complete viral inhibition at 200 nM, with the EC_50_ of 12.4 nM against Beta variant and below 6.9 nM against other three variants. Similarly, apilimod showed the EC_50_s below 6.9 nM to all tested variants. These data revealed that PIKfyve inhibitors exhibited much better inhibition with EC_50_s of 10–100 fold lower compared with Remdesivir and PF-07321332, respectively (Fig. 1d and Supplementary Fig. S3a). The remarkable antiviral activities of PIKfyve inhibitors were consistent with the data in our pseudovirions assay (Supplementary Fig. S3b and Table S2-S3). In addition, we treated Vero E6 cells for 48 h in cytopathic effect (CPE) assays (Supplementary Fig. S4a). Results showed that XMU-MP-7 and apilimod significantly rescued the cytopathic effects caused by SARS-CoV-2 and its variant Omicron (Supplementary Fig. S4b). Similarly, PIKfyve inhibitors also blocked the cytopathic effect induced by Omicron in A549, and their anti-viral activities were better than that of Remdesivir and PF-07321332 (Supplementary Fig. S4c). Together, these results reveal that SARS-CoV-2 and its variants are much more sensitive to PIKfyve inhibitors.

PIKfyve plays a critical role in endocytosis that is often hijacked by virus for host cells entry.^2^ Here, we employed a genetically engineered sensor of fluorescent protein (Gamillus)-fused SARS-CoV-2 spike trimer (STG) to probe the dynamic virus entry and explore how PIKfyve inhibitors may affect this process.^5^ Three hours after incubation, the STG probes could be internalized and observed as green dots in cytoplasmic region. However, upon treatment with XMU-MP-7 or apilimod, STG probes were almost completely trapped on the enlarged cytoplasmic vacuoles and colocalized with internalized ACE2-mRuby to form yellow fluorescent plaques. In contrast, when treated with cmpd 24 or remdesivir, STG entered the cytoplasm and emitted punctate green fluorescence similarly to that of the control group (Fig. 1e). Quantitative characterizations of STG-internalization demonstrated that PIKfyve inhibitors induced a decrease in internalized vehicle numbers (IVNs) and an increase in internalized vehicle area (IVA) in a dose-dependent manner (Supplementary Fig. S5).^5^ This data indicates that PIKfyve inhibitors may block the viral/cell membrane fusion stage, resulting in failure of viral ssRNA to be released into the cytoplasm. The viral/cell membrane fusion should be proteolytically activated by host cell proteases including cathepsin B/L.^6^ It has been recently reported that PIKfyve inhibitor apilimod inhibits cathepsin class of lysosomal proteases,^7^ which is consistent with our observation that XMU-MP-7 impaired the maturation of active cathepsin B in time- and dose- dependent manners (Fig. 1f). Collectively, PIKfyve inhibitors terminate cell entry of SARS-CoV-2 by blocking viral/cell membrane fusion.

Moreover, our time-of-addition experiment further indicated that PIKfyve inhibitors functioned at both entry and post-entry stages of the SARS-CoV-2 infection, strongly inhibiting viral replication (Fig. 1g) and the expression of viral nucleoprotein (Fig. 1h). By comparison, adenosine analogue remdesivir only exerted an inhibitory effect at post-entry stage, which was consistent with its putative antiviral mechanism by hindering viral RNA replication. We noticed that both XMU-MP-7 and apilimod induced visible cytoplasmic vacuoles within a very short time (less than 20 min), which may contribute to their antiviral activity in post-entry stage. Even if a certain degree of virus got entry before drug treatment, the rapidly generated vacuoles and inactive cathepsins induced by PIKfyve inhibitors will seriously impair the new lifecycle of virus and consequently reduce infection. Furthermore, vacuolization could be maintained for up to 48 h or longer even when drug was withdrawn after only 4 h of treatment (Fig. 1i). The remaining vacuoles appeared to be sufficient to inhibit viral infection.

In summary, we demonstrate a novel PIKfyve inhibitor XMU-MP-7 effectively overrides SARS-CoV-2 and its variants including the most concerned Delta and Omicron in vitro. Moreover, XMU-MP-7 prevents SARS-CoV-2 from entering the cytoplasm and plays potent antiviral roles at both entry and post-entry stages. The strong antiviral potency makes XMU-MP-7 as a good starting-point for developing antiviral agent against the current pandemic. Our findings support that pharmacological targeting PIKfyve to intervene the host cells’ endocytosis is an efficient way to block viral infection.

## Supplementary information

Supplemental material

## Data Availability

All data are available upon request from the corresponding author.
