# Supplementary material for: PIKfyve inhibitors against SARS-CoV-2 and its variants including Omicron
Source: Signal Transduct Target Ther. 2022 May 24;7:167. doi: 10.1038/s41392-022-01025-8 (PMC9127493; doi:10.1038/s41392-022-01025-8)
Supplement: Supplementary file 1 — Supplemental material [file 41392_2022_1025_MOESM1_ESM.docx]

Supplementary materials for

**PIKfyve inhibitors against SARS-CoV-2 and its variants including Omicron**

Jingyi Su^1, #^, Jing Zheng^2,#^, Wei Huang^1, #^, Yali Zhang^3,#^, Cairui Lv^1^, Baoding Zhang^1^ Lina Jiang^2^, Tong Cheng^3^, Quan Yuan^3^, Ningshao Xia^3^, Jianming Zhang^4^,Li Li^2,*^, Li Li^1,*^, Xianming Deng^1,*^

Correspondence: Li Li (strlchen@163.com), or Li Li (lli@xmu.edu.cn) or Xianming Deng (xmdeng@xmu.edu.cn)

**This PDF file includes:**

Materials and Methods

Supplementary Figures S1-5

Supplementary Tables S1-3

**Materials and Methods**

**Cell lines and viruses**

African green monkey kidney Vero E6 cell line, human non-small cell lung cancer cell line A549, human embryonic kidney cell line 293, 293T stably expressing recombinant human ACE2 were maintained in DMEM supplemented with 10% FBS and 1% penicillin/streptomycin. All cell lines were maintained in a humidified CO_2_ incubator at 37℃, and were thawed and passaged at least 3 times before initiating experiments. SARS-CoV-2 and its four variants including Alpha, Beta, Delta and Omicron were isolated from clinical-confirmed COVID-19 patients in Xiamen Center for Disease Control and Prevention. All viruses were propagated in Vero E6 cells, and viral titer was determined by 50% tissue culture infective dose (TCID50) using Reed-Muench assay. All the infection experiments were conducted at Bio-safety Level 3 laboratory.

**Reagents**

Apilimod (cat.# BD449971), Remdesivir (cat.# BD766429) were purchased from Bidepharm, and Chloroquine (cat.# 109M4003V) was purchased from Sigma. PF07321332 (cat.# LL-1323701LL) was purchased from Lollane. XMU-MP-7 and cmpd 24 were chemically synthesized by the laboratory.

**Morphological observations**

Cells were seeded into 96-well plates and treated with test drugs for indicated time periods. Pictures of cells were subsequently obtained using Biotek Cytation-5&BioSpa8.

**Antiproliferation assay**

Cells were seeded into 96-well plates and exposed to serially diluted drugs for 48 h. Cell viability was determined using MTS (Promega, Madison, WI, USA), according to the manufacturer’ s instructions. The absorbance was measured in Varioskan Flash (Thermo) at 490 nm. Data were normalized to the control group (DMSO). CC_50_ values were calculated by concentration-response curve-fitting using GraphPad Prism.

**Antiviral efficacy assay**

Cells were seeded into 96-well plates and cultured overnight in complete medium. Cells were pre-treated with serially diluted drugs for 2 h, and the virus (MOI of 0.05) was subsequently added. At 2 h p.i., the virus-drug mixture was removed and cells were washed twice with PBS before being further cultured in fresh drug-containing medium. 48 hours after infection, 80 microliter cell culture supernatant was collected and lysed with High Pure Viral RNA Kit (Roche, cat.# 11858882001) to extract viral RNA. Finally, the viral RNA was dissolved in 50 μL RNase-free water. cDNA was sythezed by Hifair® Ⅲ 1st Strand cDNA Synthesis SuperMix (Yeasen, cat.# 11141ES10) and then used as template for quantitative PCR (Yeasen, cat.# 11201ES03). Primers used in quantitative PCR were RBD-qPCR-F: 5’-CAATGGTTTAACAGGCACAGG-3’ and RBD-qPCR-R: 5’-CTCAAGTGTCTGTGGATCACG-3’. A standard plasmid was constructed by cloning receptor binding domain (RBD) of spike gene into a pCMV vector. Primers were used as belows: RBD-F: 5’-AAAGTCGACGGCCTAATATTACAAACTTGTGCC-3’ and RBD-R: 5’-AAAGGTACCCTCAAGTGTCTGTGGATCAC-3’. Standard curve was generated by serially diluting the plasmid (10^3^-10^9^ copies) and determining the corresponding cycle threshold. According to the standard curve, the copies of each sample were calculated and the correlation curve between copies and drug concentration was finally formulated.

**Cytopathic effect (CPE) inhibition assay**

The cell monolayers were grown in 96-well plates and treated with different drugs for 2 h before virus infection. Then cells were infected with SARS-CoV-2 or Omicron variant at 37℃ for 2 h in the presence of drugs. The drug-virus mixture was then removed, and cells were subsequently incubated with indicated concentrations of drugs. Following 48 h of incubation, the images of cytopathic effect were obtained.

**Time-of-addition experiment**

Vero E6 cells were plated into 6-well plates and grew overnight. During “Full-time” treatment, cells were first treated with drug for two hours, and then infected with the virus for two hours. After that, the mixed supernatant was removed and cells were washed twice with PBS and finally cultured in fresh medium containing the same concentration of drug. For “Entry” treatment, pre-treatment and infection operations were similar to that in “Full-time” treatment. However, cells were cultured in drug-free medium after the mixed supernatant removed. As for “Post-entry” treatment, cells were infected with virus for 2 h before treated with drug for 2 h. Then the mixture was removed and fresh medium containing drug was added after cells were washed with PBS. The DMSO-control group was treated in the same way as “Full-time” group. For quantitative PCR, the MOI was controlled at 0.05 and supernatant was harvest at 24 h post infection. While for western blot, MOI was controlled at 0.30 and cells were harvest at 72 h post infection to identify the virus protein expression.

**Western blot analysis**

After gently washed with ice-cold PBS, cells were lysed in lysis buffer (1% Triton X-100, 150 mM NaCl, 50 mM Tris-HCl, 5% glycerin) containing protease inhibitors and phosphatase inhibitors. For immunoblotting, protein samples were separated on 10% SDS-PAGE and then electrically transferred to a PVDF film. After blocked with 1×TBS containing 5% BSA, the film was incubated with primary antibody, followed by HRP-conjugated secondary antibody. Protein bands were finally detected by the enhanced chemiluminescence reagent, according to the manufacturer’s instructions (Advansta, CA, USA). Primary antibodies against GAPDH (cat.#2118L), cathepsin B (cat.#31718), anti-rabbit or anti-mouse IgG horseradish peroxidase (HRP)-linked secondary antibodies were purchased from Cell Signaling Technology. Primary antibodies against SARS Nucleocapsid (cat.# ab273434) was purchased from Abcam. Primary antibodies against α-tubulin (cat.# T6074) was purchased from Sigma. All primary antibodies were uniformly pre-diluted in TBS-T at 1:1000 (v/v).

**Visulization of SARS-CoV-2 cell entry**

To visualize the effect of drugs on viral entry, 293T-ACE2iRb3 cells were pretreated with serially diluted drugs for 1 h before STG probes addition for further incubation. Cell images shown in **Fig 1e** were acquired using a 100× oil immersion objective. The data shown in **Fig S3** was derived from images acquired on Opera Phenix using 40× water immersion objective. For images of **Fig 1e**, cells were gently washed twice with PBS after 3 h incubation with STG, following a paraformaldehyde fixation before imaging. Cell images involved in **Fig S3** were obtained under wash-free and live-cell conditions. The construction of STG probe used here and the image quantitative analysis were performed as previously described^1^.

**Production of SARS-CoV-2 S pseudovirions**

The codon-optimized gene of SARS-CoV-2 S glycoprotein with C-terminal 18 amino acids deletion was synthesized and cloned into pcDNA3.3 between BamHI and Xho I. The plasmid mentioned above and the pBOBI lentiviral reporter plasmid which expresses firefly-luciferase were both generously gifted from Changchun Xiao, Xiamen University. pcDNA3.3-SARS-CoV-2 S was used as a template for point mutation to construct plasmids with mutations at different sites. pcDNA3.1-SARS-CoV-2 S (Omicron-32 mutations in full length spike) was purchased from GenScript, while the pcDNA3.3-SARS-CoV-2 S (Omicron-15 mutations in RBD region)-△C18 was constructed later. 293T cells were co-transfected with pMDL, pRSV-Rev, pBOBI-luciferase and pcDNA3.3-SARS-CoV-2 S by using PEI to generate pseudovirions. The supernatants were harvested at 72 h post transfection and centrifuged at 500 × g for 10 min to remove cell debris^2^.

**Determination of Pseudovirions infectivity**

293T-hACE2 cells were seeded into 96-well plate and treated with serially diluted drugs for 2 h before transduced with pseudovirions. After 48 h incubation in drugs-pseudovirion mixture, cells were lysed on ice and the luciferase activity was measured using Varioskan Flash. EC_50_ values were then calculated by concentration-response curve-fitting using GraphPad Prism.

***K*ds determination**

KINOMEscan technology is a competitive affinity-based binding assay provided by Eurofins DiscoverX Corporation^3^. Briefly, kinases were produced in HEK-293 cells and tagged with DNA for qPCR detection. Affinity resins were generated by treating streptavidin-coated magnetic beads with biotinylated small molecule ligands. For binding assays, the liganded beads were combined with DNA-tagged kinases and the test compounds. DMSO was used as a control. Compounds that bind to the DNA-tagged kinases will prevent these kinases binding with immobilized ligands and reduce the amount of the corresponding kinases captured on solid support. *K*ds were determined using 11 serial threefold dilutions of the test compounds and a DMSO control.

**Molecular modeling study**

Molecular docking studies was performed using the Glide program ^4^. The structure of PIKfyve kinase domain was extracted from PIKfyve/Fig4/Vac14 complex (PDB code: 7k2v) as the template ^5^.

**Statistical analysis**

Data were represented as mean ± SEM of at least three independent experiments. Comparisons between two groups were analyzed using the 2-tailed Student’s *t* test by GraphPad Prism software. P value < 0.05 was considered as statistically significant.

**References**

1 Zhang, Y. *et al.* Virus-free and live-cell visualizing SARS-CoV-2 cell entry for studies of neutralizing antibodies and compound inhibitors. *Small Methods* **5**, 2001031 (2021).

2 Ou, X. *et al.* Characterization of spike glycoprotein of SARS-CoV-2 on virus entry and its immune cross-reactivity with SARS-CoV. *Nat. Commun.* **11**, 1620 (2020).

3 Davis, M. I. *et al.* Comprehensive analysis of kinase inhibitor selectivity. *Nat. Biotechnol.* **29**, 1046-1051 (2011).

4 Friesner, R. A. *et al.* Extra precision glide: docking and scoring incorporating a model of hydrophobic enclosure for protein-ligand complexes. *J. Med. Chem.* **49**, 6177-6196 (2006).

5 Lees, J. A., Li, P., Kumar, N., Weisman, L. S. & Reinisch, K. M. Insights into Lysosomal PI(3,5)P2 Homeostasis from a Structural-Biochemical Analysis of the PIKfyve Lipid Kinase Complex. *Mol. Cell* **80**, 736-743 (2020).


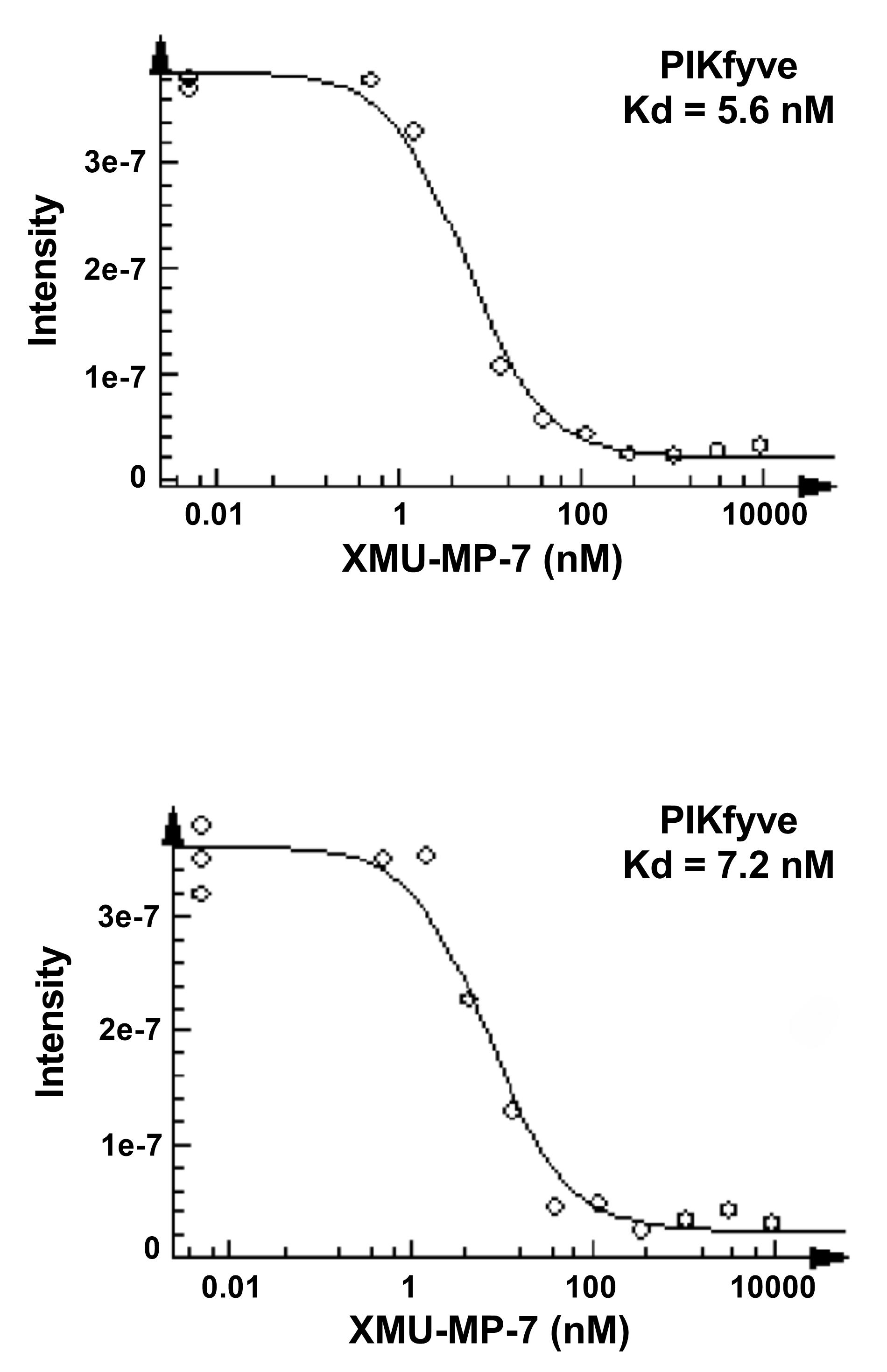

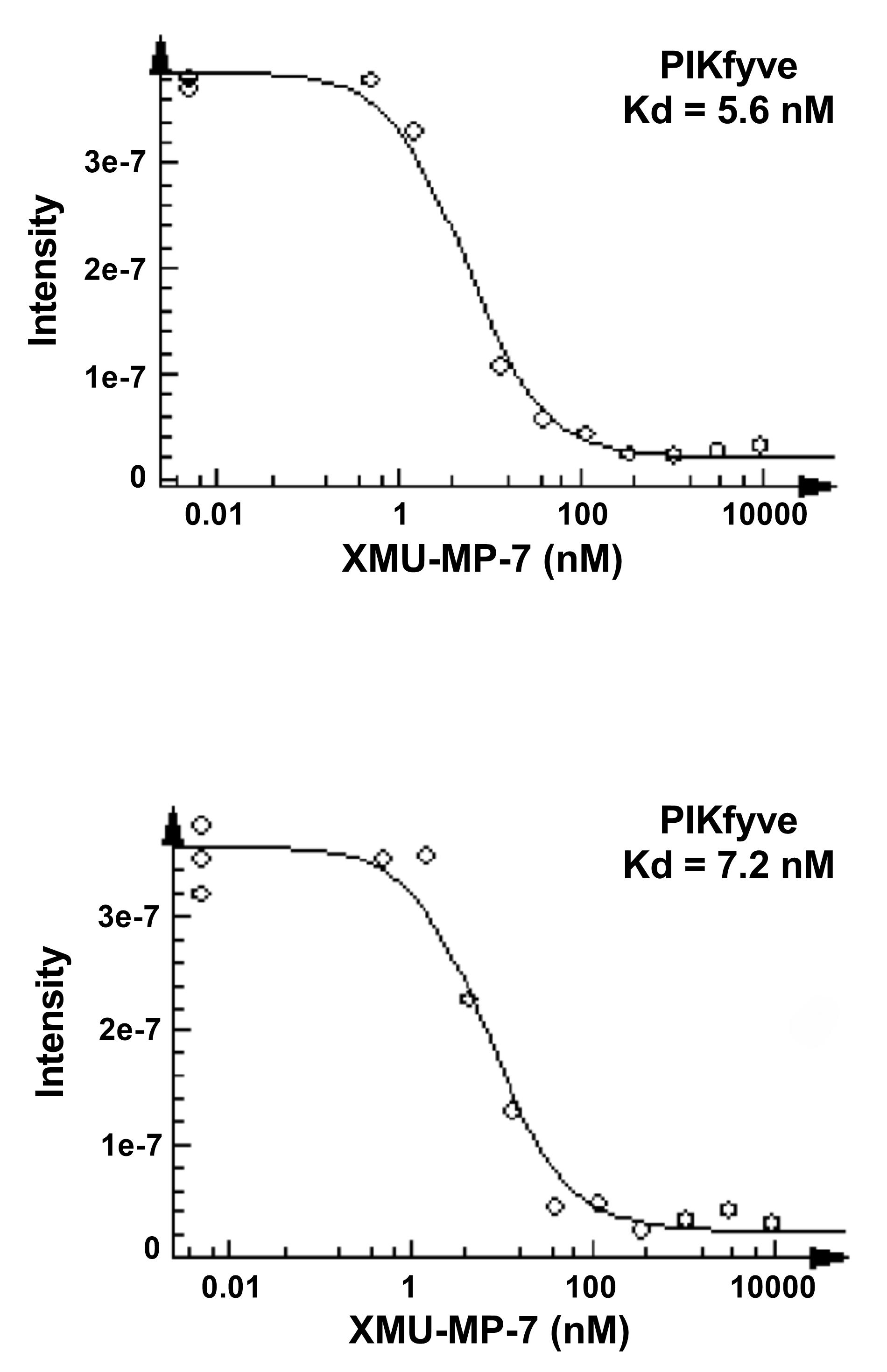


**Supplementary Figure S1. The equilibrium dissociation constant (*K*d) for XMU-MP-7 with PIKfyve was determined from 2 independent titration curves.**

**
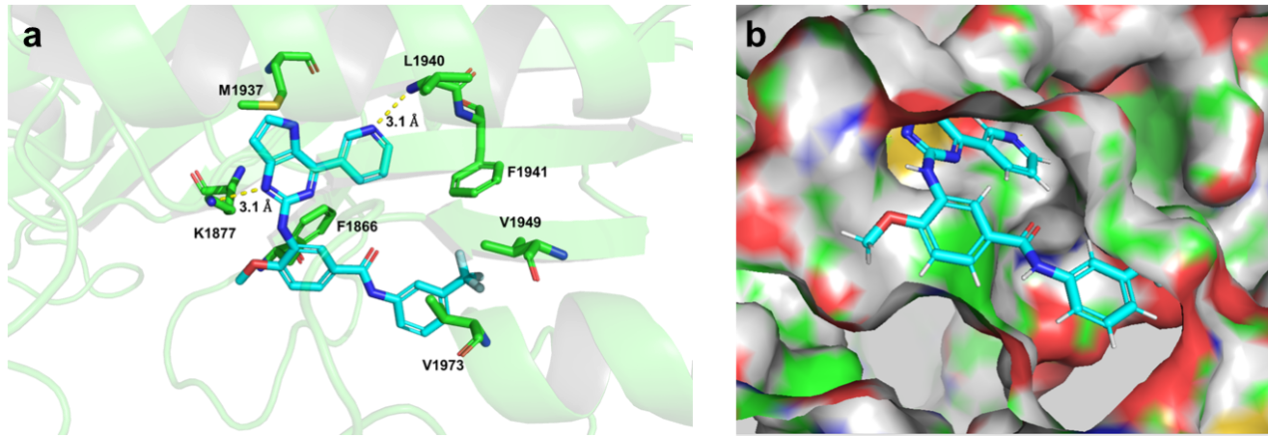
Supplementary Figure S2.** **Molecular modeling of XMU-MP-7 binding mode.**

**a** Binding mode of XMU-MP-7 with PIKfyve.

**b** The surface view of binding pocket of XMU-MP-7 with PIKfyve. The structure of PIKfyve kinase domain was extracted from PIKfyve/Fig4/Vac14 complex (PDB code:7k2v) as the template.

**Note:** The molecular docking study revealed that XMU-MP-7 (cmpd 38) binds to the PIKfyve kinase domain in a non-canonical binding mode. The nitrogen atom of the pyrimidine ‘core’ moiety forms a hydrogen bond with residue Lys1877 within the distance of 3.1 Å. The pyridin-3-yl nitrogen atom forms a hydrogen bond with residue Leu1940 as well. Additionally, the trifluoromethyl substituted phenyl ring occupies the hydrophobic pocket created by Phe1941, Val1949 and Val1973. However, comparing with cmpd 38, cmpd 24 with pyridine-4-yl substituent, loses the hydrogen interaction with Leu1940 and introduces steric clash, which leads to its great loss of PIKfyve inhibition activity.


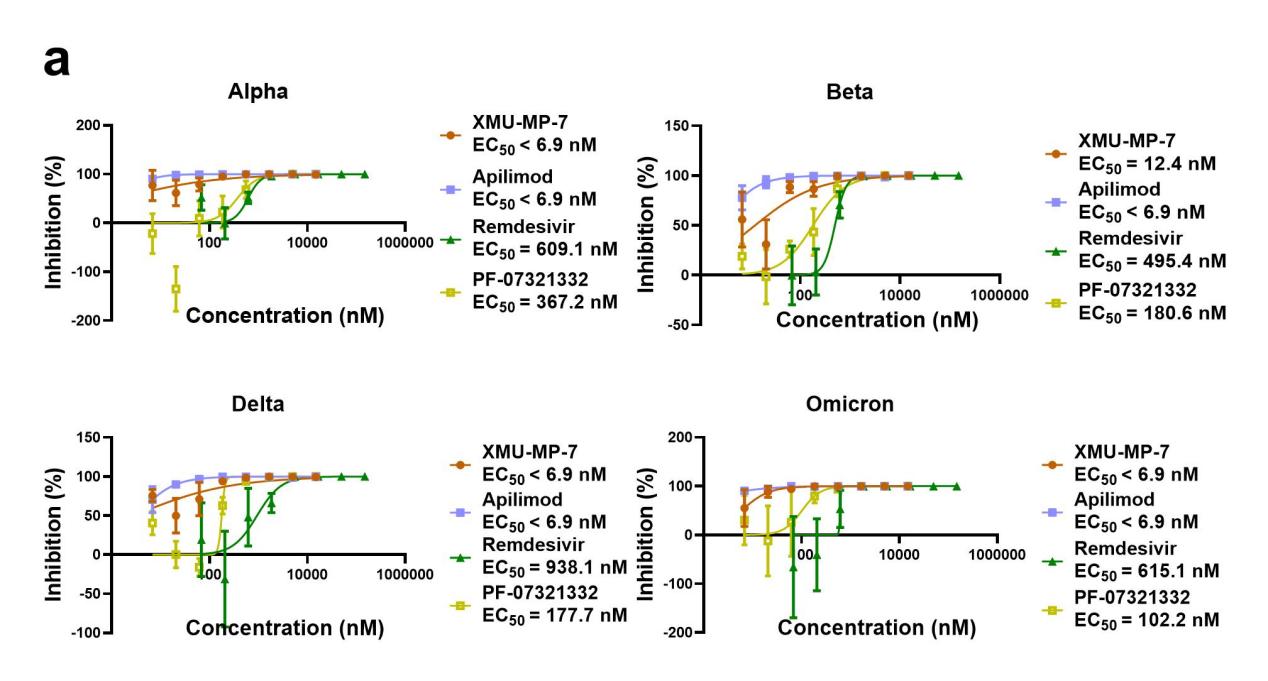


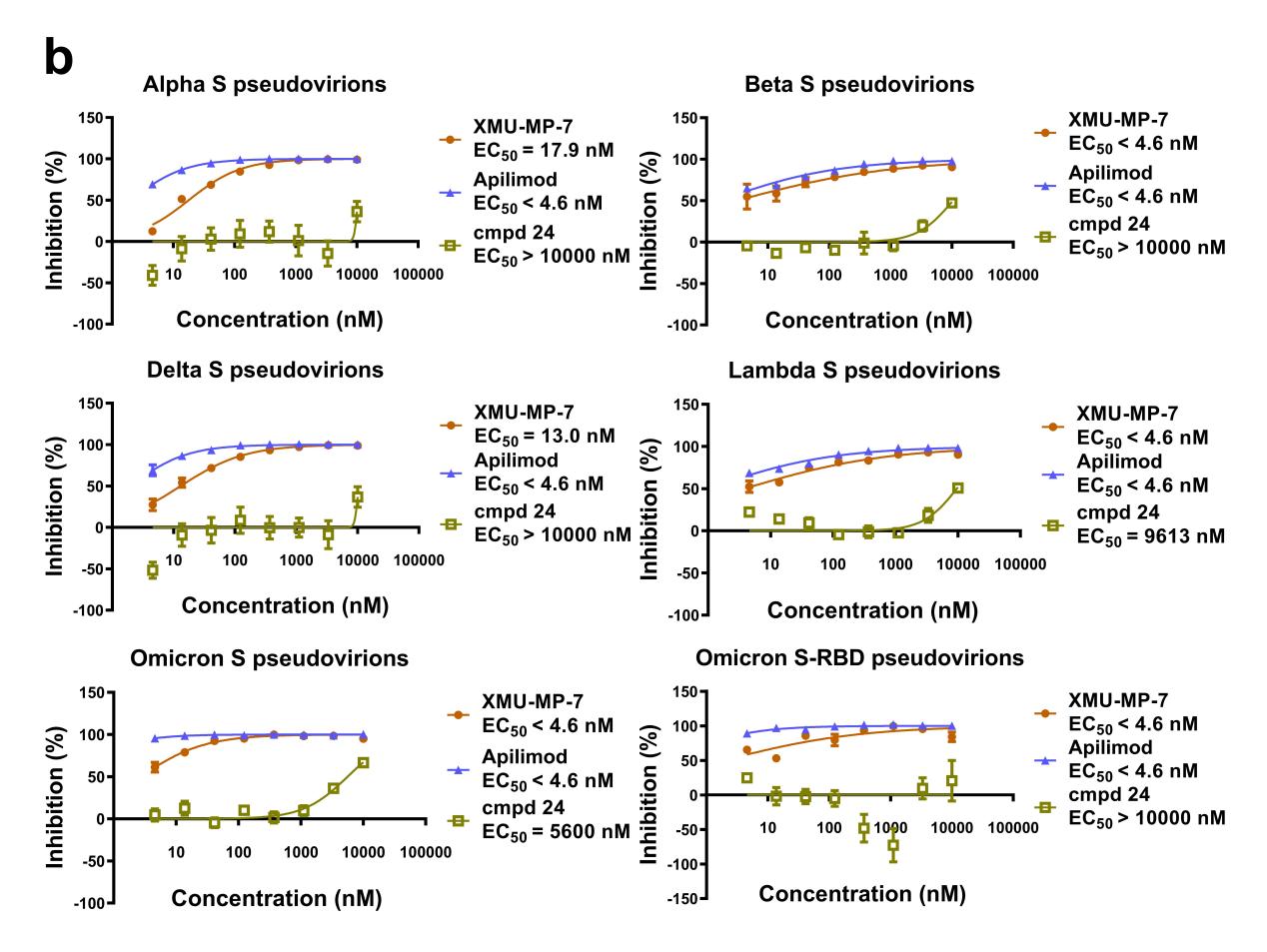


**Supplementary Figure S3. The dose response curves of selected drugs against SARS-CoV-2 variants.**

**a** The dose response curves of selected drugs against four SARS-CoV-2 variants of concern (n = 6). Graphs were represented as means ± SEM.

**b** Antiviral activities against multiple pseudoviruses that mimicked five novel SARS-CoV-2 variants (n = 3). The experiments were conducted in duplicate.


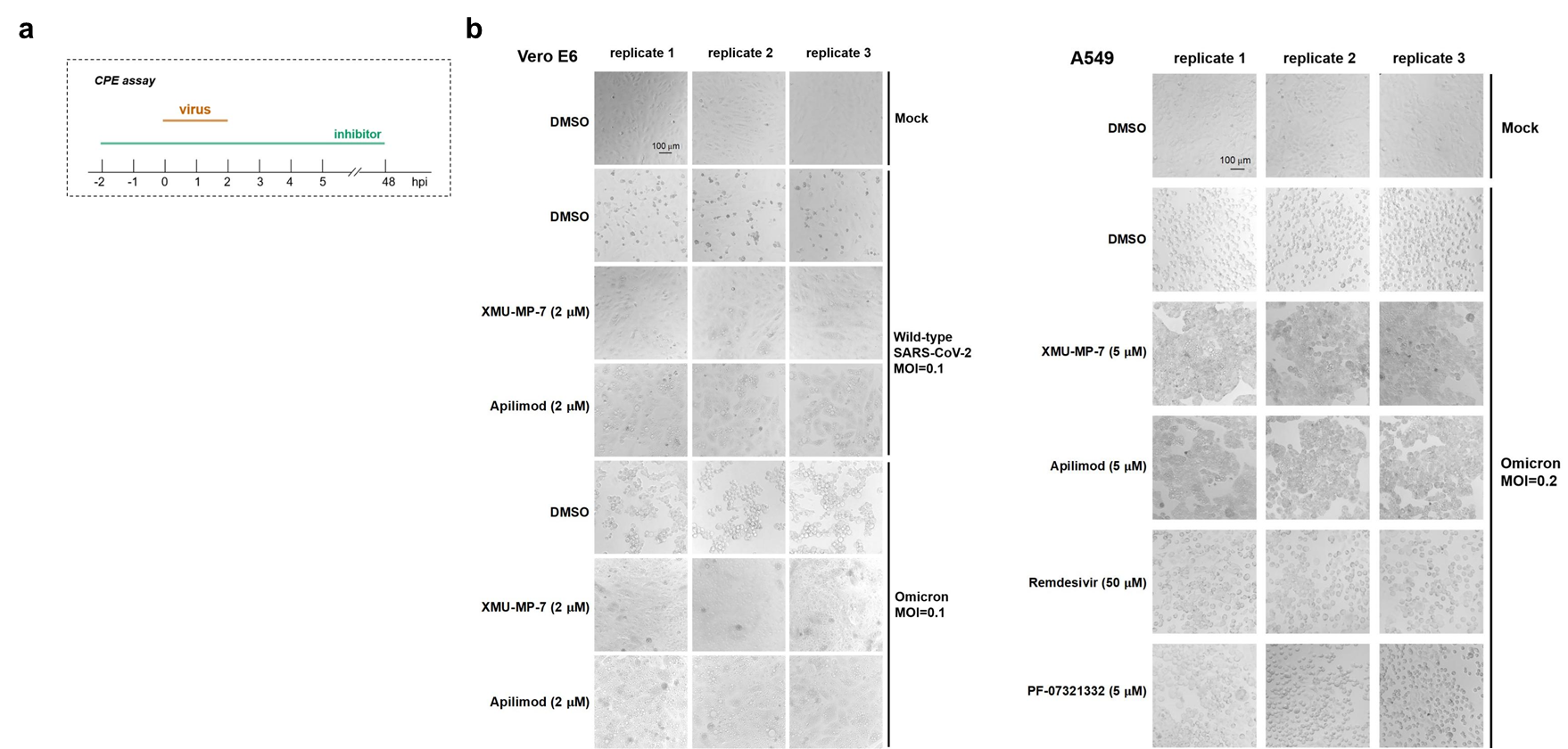


**Supplementary Figure S4. Virus-induced cytopathic effect in Vero E6 and A549 cells after PIKfyve inhibitors treatment.**

**a** Schematic of cytopathic effect (CPE) assay.

**b** Virus-induced cytopathic effect of wild-type SARS-CoV-2 and Omicron in Vero E6 cells (MOI = 0.1).

**c** Virus-induced cytopathic effect of Omicron in A549 cells (MOI = 0.2).

**
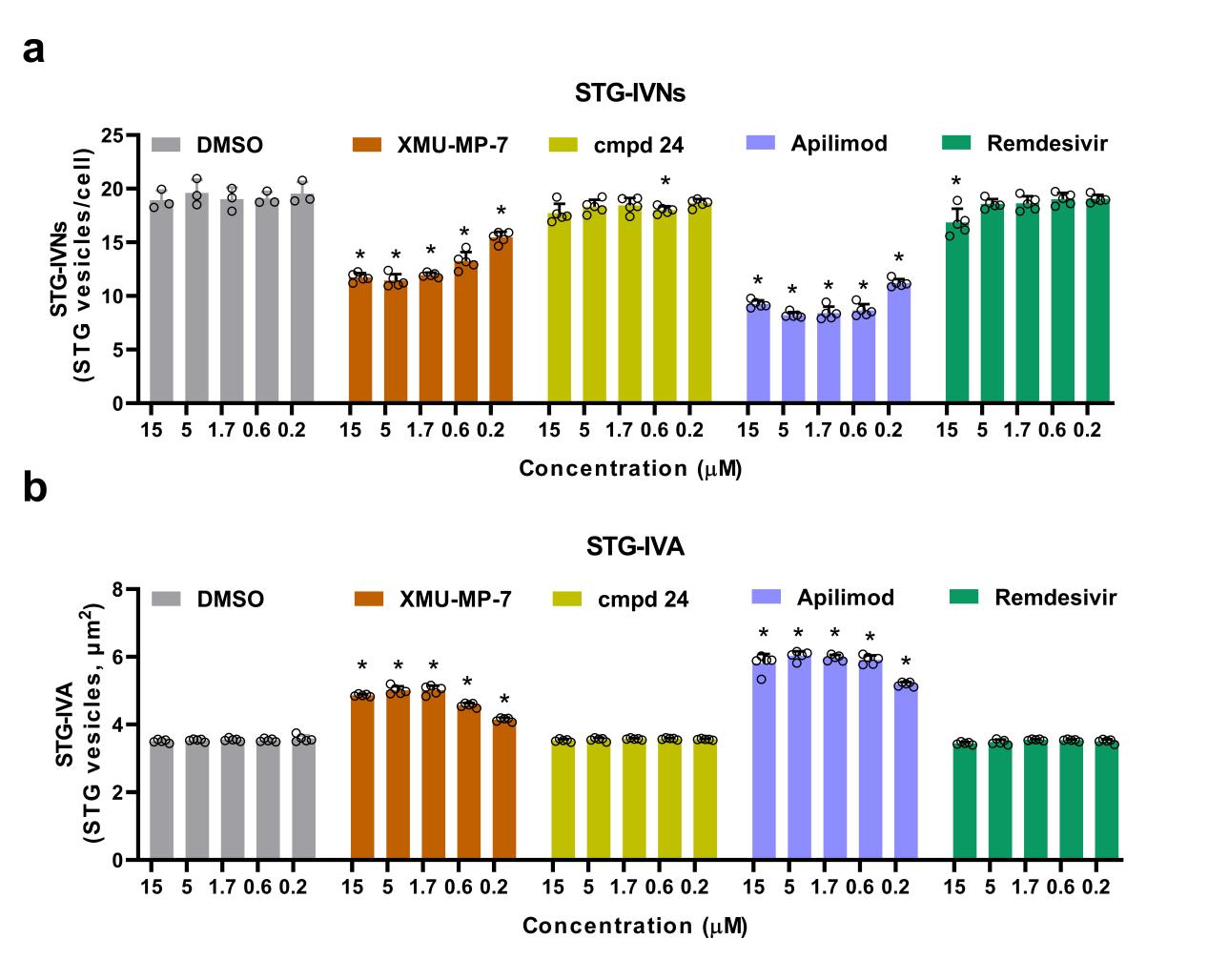
**

**Supplementary Figure S5. Quantitative analysis of STG internalization upon drugs treatment.**

All drugs were tested in a 3-fold dilution series and the initial drug concentration was 15 μM. Three and five replicate wells were performed for the DMSO and drug groups, respectively. 25 fields of each well were imaged for quantitative analyses after 3 h incubation with STG-probe.

**a** STG-IVNs, average numbers of internalized STG vesicles per cell;

**b** STG-IVA, average area (µm^2^) of internalized STG vesicles. P values were calculated by unpaired t-test. *p < 0.05.

**Supplementary Table S1. The pharmacokinetic properties of XMU-MP-7 in rat.**

|  | **Dose** | **T_1/2_** | **T_max_** | **C_max_** | **C_0_** | **AUC_(0-t)_** | **AUC_(0-∞)_** | **V_d_** | **Cl** | **MRT_(0-∞)_** | **F** |
| --- | --- | --- | --- | --- | --- | --- | --- | --- | --- | --- | --- |
| **Route** | **(mg/kg)** | **hr** | **hr** | **ng/mL** | **ng/mL** | **ng/mL*****hr** | **ng/mL*****hr** | **L/kg** | **L/hr/kg** | **hr** | **%** |
| IV | 2 | 0.608 | 0.08 | - | 13981 | 4251 | 4254 | 0.20 | 7.88 | 0.429 | - |
| PO | 10 | 3.06 | 2.33 | 429 | - | 2200 | 2213 | - | - | 3.81 | 10.4 |

The pharmacokinetics of XMU-MP-7 were determined following single intravenous and oral administration in Sprague Dawley rat (N = 3/each) at the dose indicated. Blood samples were collected at 0.08, 0.25, 0.5, 1, 2, 4, 8 & 24 hr (IV) & 0.25, 0.5, 1, 2, 4, 6, 8 & 24 hr (PO) post dose. Then the samples were quantified by LC-MS/MS, and data analysis was conducted using WinNonlin V6.3. IV = intravenous injection, PO = oral delivery, T_max_ = time of maximum plasma concentration, C_max_ = maximum plasma concentration, AUC = area under the curve (measure of exposure), T_1/2_ = half life, Cl = plasma clearance, V_d_ = volume of distribution, F = oral bioavailability.

**Supplementary Table S2. Key mutations of novel SARS-CoV-2 variants constructed in different pseudoviruses.**

| **WHO nomenclature** | **Lineage** | **Designation** | **Status** | **Important mutation sites** |
| --- | --- | --- | --- | --- |
| Alpha | B.1.1.7 | VOC-20DEC-01 | VOC | 69-70 HV deletion, N501Y, D614G |
| Beta | B.1.351 | VOC-20DEC-02 | VOC | K417N, E484K, N501Y |
| Delta | B.1.617.2 | VOC-21APR-02 | VOC | L452R, E484Q |
| Lambda | C.37 |  | Monitoring | T76I, L452Q, F490S |
| Omicron | B.1.1.529 | VOC-21NOV-01 | VOC | 32 mutations in full-length spike |
|  |  |  |  | 15 mutations in spike RBD region |

pcDNA3.3-SARS-CoV-2 spike was used as a template for point mutation to construct plasmids with mutations at different sites. The last column of table displayed the corresponding mutations constructed on pseudoviruses which mimicked the different SARS-CoV-2 variants.

**Supplementary Table S3. The cytotoxicity of drugs used in pseudovirus experiments.**

| **Cmpd ID** | **CC_50_ (nM)** |
| --- | --- |
| XMU-MP-7 | > 10000 |
| Apilimod | > 10000 |
| cmpd 24 | > 10000 |

293T-hACE2 cells were treated with serially diluted drugs and cytotoxicity was determined by MTS assay at 48 hours (n = 3).
